# Adding carbon fiber to shoe soles may not improve running economy: a muscle-level explanation

**DOI:** 10.1038/s41598-020-74097-7

**Published:** 2020-10-13

**Authors:** Owen N. Beck, Pawel R. Golyski, Gregory S. Sawicki

**Affiliations:** 1 George W. Woodruff School of Mechanical Engineering, Georgia Institute of Technology, Atlanta, GA USA; 2 School of Biological Sciences, Georgia Institute of Technology, Atlanta, GA USA; 3 Parker H. Petit Institute for Bioengineering and Biosciences, Georgia Institute of Technology, Atlanta, GA USA

**Keywords:** Metabolism, Anatomy

## Abstract

In an attempt to improve their distance-running performance, many athletes race with carbon fiber plates embedded in their shoe soles. Accordingly, we sought to establish whether, and if so how, adding carbon fiber plates to shoes soles reduces athlete aerobic energy expenditure during running (improves running economy). We tested 15 athletes as they ran at 3.5 m/s in four footwear conditions that varied in shoe sole bending stiffness, modified by carbon fiber plates. For each condition, we quantified athlete aerobic energy expenditure and performed biomechanical analyses, which included the use of ultrasonography to examine soleus muscle dynamics in vivo. Overall, increased footwear bending stiffness lengthened ground contact time (p = 0.048), but did not affect ankle (p ≥ 0.060), knee (p ≥ 0.128), or hip (p ≥ 0.076) joint angles or moments. Additionally, increased footwear bending stiffness did not affect muscle activity (all seven measured leg muscles (p ≥ 0.146)), soleus active muscle volume (p = 0.538; d = 0.241), or aerobic power (p = 0.458; d = 0.04) during running. Hence, footwear bending stiffness does not appear to alter the volume of aerobic energy consuming muscle in the soleus, or any other leg muscle, during running. Therefore, adding carbon fiber plates to shoe soles slightly alters whole-body and calf muscle biomechanics but may not improve running economy.

## Introduction

In competitive athletics, marginal differences distinguish champions from their competitors. For instance, if any of the top-five 2016 Olympic women’s marathon finishers ran 0.51% faster, they would have been crowned Olympic champion. Such miniscule differences highlight the importance for athletes to optimize all factors that influence race performance. One way to optimize athletic performance is to don the best footwear. Using footwear that reduces athlete aerobic energy expenditure at a given running speed (improves athlete running economy) can augment distance-running performance by decreasing user relative aerobic intensity^1–3^. An established method of improving footwear to augment athlete distance-running performance is to reduce its mass^1,2,4,5^. Based on literature values, if an aforementioned Olympic marathoner re-raced in shoes that were 100 g less than their original footwear, they would have expended aerobic energy at an ~ 0.8% slower rate^5^, run the marathon ~ 0.56% faster^6^, and taken the gold medal back to their country.

A longstanding footwear technology that has polarized the running community is the incorporation of carbon fiber plates in shoe soles^7^. Despite the rampant use of carbon fiber plates in athletics^8–10^, policy makers are regulating the use of these plates in distance-running footwear based on the notion that they provide wearers an ‘unfair advantage’ over competitors without such technology^11^. These views persist even though it is inconclusive whether adding carbon fiber to shoe soles improves running economy^12–16^ or distance-running performance. To date, two studies have reported that adding optimally stiff carbon fiber plates to shoe soles improves running economy by 0.8^12^ and 1.1%^13^, while *data* from four other studies suggest that adding carbon fiber plates to shoe soles does not affect running economy^14–17^.

Moreover, neither study that improved athlete running economy by adding carbon fiber plates to their shoes measured a physiologically-relevant link between the footwear-altered biomechanics and aerobic energy expenditure^12,13^. Namely, the first study did not identify a biomechanical mechanism^12^ while the second study suggested that adding carbon fiber plates to shoe soles improves running economy by altering a parameter that likely does not affect metabolism^13^. Specifically, the second study reported that adding carbon fiber plates to shoe soles improves running economy by decreasing the leg-joint’s summed angular impulse (integral of torque with respect to time) during push-off^13^. However, decreasing angular impulse via greater peak torque and much shorter durations worsen running economy^18–20^. Consequently, it remains uncertain whether adding carbon fiber plates to shoe soles improves running economy, and if so how—we need a muscle-level explanation.

Muscle contractions drive whole-body aerobic energy expenditure during locomotion^21^. To date, no study has assessed muscle fascicle dynamics from athletes running with shoes that have carbon fiber soles. Based on leg-joint analyses, which do not necessarily reflect the underlying fascicle dynamics^22,23^, metatarsophalangeal- and ankle-joint dynamics are more affected during running with the addition of carbon fiber plates to shoe soles than knee- and hip-joint dynamics^13,14,24–26^. Since intrinsic foot muscles do not directly affect running economy^27^, altered plantar flexor fascicle dynamics may help explain changes in running economy with versus without carbon fiber plates added to shoe soles.

How does adding carbon fiber plates to shoe soles affect athlete plantar flexor dynamics during running? Adding carbon fiber plates to shoe soles increases the footwear’s 3-point bending stiffness^12,13,15,17,24,25,28^ and typically shifts the athlete’s center of pressure more anterior along the foot during ground contact^24,25,28,29^. These altered biomechanics generally yield a longer moment arm between the ground reaction force (*F*_*GRF*_) and the ankle-joint center ($R_{GRF}$)^13,24^. Longer moment arms lead to greater GRF-induced ankle-joint moments^12,13,24,29^. To prevent the ankle-joint from collapsing, plantar flexor muscle-tendons (MTs) need to generate a greater force ($F_{MTs}$) and apply an equal and opposite moment about the joint throughout ground contact.

1 $$r_{MTs} \cdot F_{MTs} = R_{GRF} \cdot F_{GRF}$$

The moment arm between the plantar flexor MTs and ankle-joint center is indicated by $$r_{MT}$$^30^. Increased MT force is driven by greater plantar flexor muscle fascicle force ($$F_{M}$$), which increases metabolic energy expenditure^31^ and can be calculated using the following (Eq. (2)): plantar flexor MT force ($$F_{MT} )$$, its physiological cross-sectional area relative to respective agonist muscles $$\left( {PCSA_{\frac{m}{tot}} } \right)$$^30^, and pennation angle ($$\theta_{M}$$).

2 $$F_{M} = \frac{{F_{MT} PCSA_{\frac{m}{tot}} }}{{\cos \left( {\theta_{M} } \right)}}$$

Adding carbon fiber plates to footwear may also cause plantar flexors to operate at relatively shorter lengths; incurring less economical muscle force production^32–35^. That is because running in footwear that have carbon fiber plates elicits similar leg-joint angles^12,13^ and MT lengths ($L_{MT}$)^36^ versus running in footwear absent of carbon fiber plates. Hence, reasoning that muscle pennation changes are relatively small, increased MT force may further stretch spring-like tendons (tendon length: $L_{T}$) and yield shorter in-series muscles lengths ($L_{M}$).

3 $$L_{M} = \frac{{\left( {L_{MT} - L_{T} } \right)}}{{{\cos}(\theta_{M} )}}$$

Lastly, adding carbon fiber plates to shoe soles may decrease plantar flexor muscle fascicle shortening velocity during ground contact^14,29^, and elicit more economical force production^33,34^. Absent of meaningful changes in ankle-joint mechanical power ($P_{ank}$) and plantar flexor MT moment arms ($r_{MTs}$), increasing plantar flexor MTs force ($F_{MTs}$) decreases ankle-joint angular velocity ($\omega_{ank}$)^14^.

4 $$\omega_{ank} = \frac{{P_{ank} }}{{r_{MTs } \cdot F_{MTs} }}$$

In turn, decreased ankle-joint angular velocity may translate to slower MT and muscle fascicle shortening velocities.

Perhaps adding carbon fiber to shoe soles can optimize the trade-off between active muscle force $(F_{act}$), force–length ($FL$) and force–velocity ($FV$) potential to minimize the active plantar flexor muscle volume ($V_{act}$)^37^ (Eq. (5)) and whole-body aerobic energy expenditure during running^12,13^. $\sigma$ is muscle stress and $l_{m}$ is optimal fascicle length.

5 $$V_{act} = \frac{{F_{act} \cdot l_{m} }}{\sigma \cdot FL \cdot FV}$$

Conceptually, active muscle volume is the quantity of muscle that has adenosine triphosphate (ATP) splitting actin-myosin cross-bridges^37^. Hence, active muscle volume is proportional to metabolic energy expenditure.

The purpose of this study was to reveal if and how adding carbon fiber plates to shoe soles alters running biomechanics and economy. In particular, we sought to investigate how footwear 3-point bending stiffness affects soleus fascicle dynamics and running economy. Based on the reported interactions between adding carbon fiber plates to shoe soles, footwear 3-point bending stiffness^12–15,17,24,25,28,29^, and ankle-joint dynamics^13,14,24,29^, we hypothesized that running with shoes that have stiffer carbon fiber plates would increase soleus fascicle force generation while decreasing its operating length and shortening velocity during the ground contact. We also hypothesized that an optimal footwear bending stiffness would minimize soleus active muscle volume and aerobic energy expenditure. To test our hypotheses, we quantified ground reaction forces, stride kinematics, limb-joint biomechanics, soleus dynamics, muscle activation patterns, and aerobic energy expenditure from 15 athletes running at 3.5 m/s using four separate footwear conditions that spanned a 6.4-fold difference in bending stiffness (Table 1).

**Table 1:** Participant characteristics.

Participant	Age (yrs)	Height (m)	Mass (kg)	Leg length (m)	US men’s shoe size	Initial foot strike	Standing aerobic power (W/kg)
1	20	1.65	57.0	0.89	9	HS	1.37
2	27	1.73	65.6	0.91	10	M/FFS	1.21
3	19	1.77	60.0	0.91	9	HS	1.98
4	27	1.88	66.4	0.97	12	M/FFS	1.44
5	27	1.70	58.9	0.83	10	HS	1.42
6	23	1.80	72.8	0.97	10	HS	1.70
7	19	1.76	71.5	0.91	10	HS	1.67
8	24	1.78	71.3	0.93	10	HS	1.58
9	20	1.80	66.5	0.95	10	HS	1.72
10	28	1.80	73.2	0.98	9	M/FFS	1.76
11	28	1.74	74.5	0.83	10	HS	1.32
12	28	1.89	75.4	0.95	12	M/FFS	1.21
13	42	1.74	73.6	0.90	11	HS	1.04
14	26	1.79	62.2	0.90	10	HS	1.38
15	23	1.78	65.2	0.89	9	HS	1.28
Average ± SD	25.4 ± 5.7	1.77 ± 0.06	67.6 ± 6.1	0.91 ± 0.05	10.1 ± 1.0	4 M/FFS 11 HS	1.47 ± 0.26

Four and eleven participants initiated ground contact with a mid/forefoot strike (M/FFS) and heel strike (HS), respectively. All participants maintained the same foot strike pattern across footwear conditions.

## Results

### Footwear conditions

Each athlete ran in the Adidas Adizero Adios BOOST 2 running shoes (Adidas) without carbon fiber plates, as well as in the Adidas with 0.8, 1.6, and 3.2 mm thick carbon fiber plates. The Adidas’ average ± SD 3-point bending stiffness was 13.0 ± 1.0 kN/m, and adding 0.8, 1.6, and 3.2 mm thick carbon fiber plates to the shoes soles increased the average ± SD footwear 3-point bending stiffness to 31.0 ± 1.5, 43.1 ± 1.6, and 84.1 ± 1.1 kN/m, respectively. Further, the slope of each footwear-condition’s 3-point bending force–displacement profile was well-characterized by a linear function (average ± SD; Adidas R^2^: 0.97 ± 0.02; Adidas plus in-soles: R^2^: 0.99 ± 0.01).

### Limb-joint dynamics

**Figure 1:**
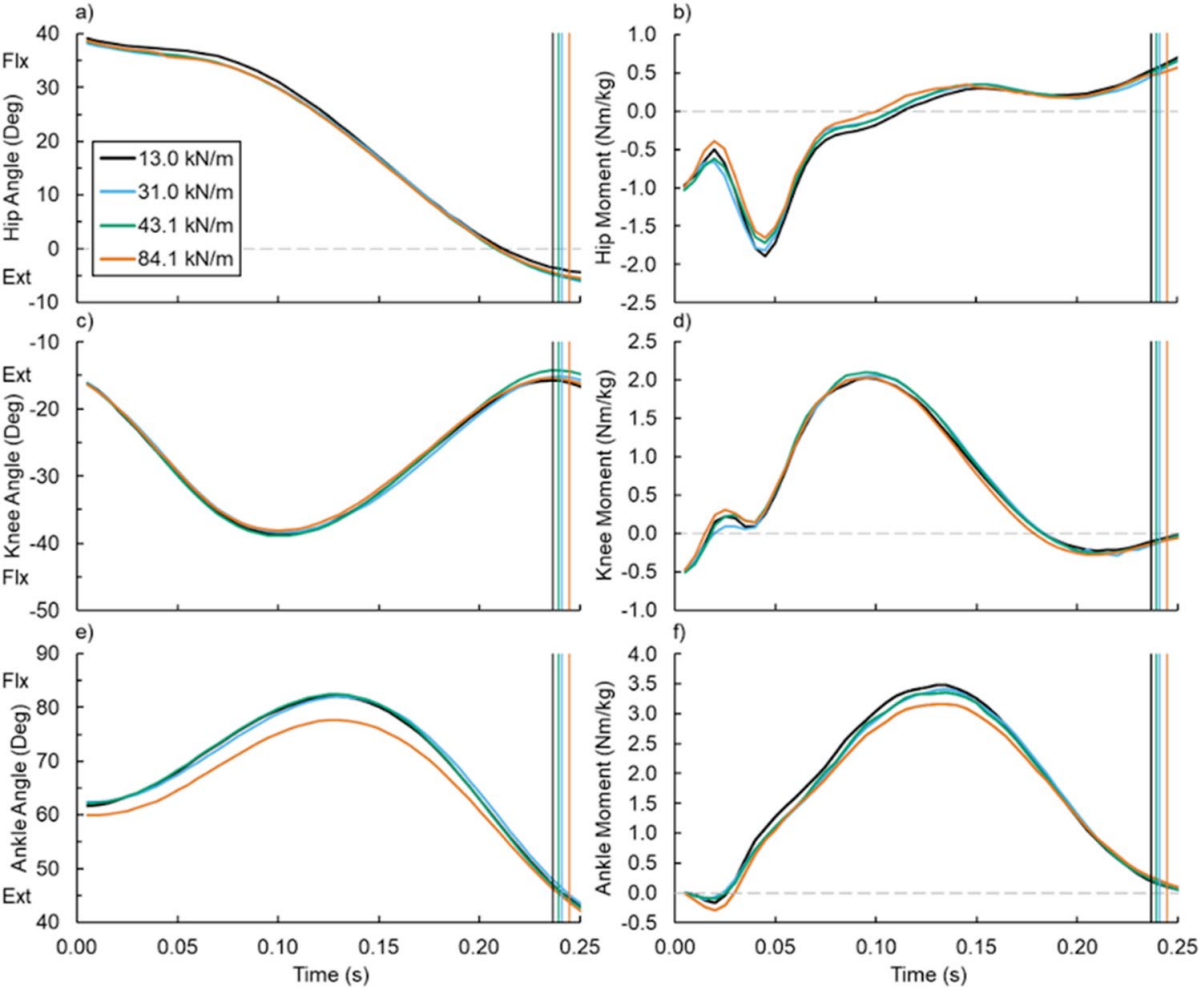
Average (**a**,**b**) hip, (**c**,**d**) knee, and (**e**,**f**) ankle angle and net moment versus time during running with footwear of varied 3-point bending stiffness: 13.0 (black), 31.0 (blue), 43.1 (green), and 84.1 kN/m (orange). Vertical lines indicate the average end of ground contact for the respective footwear condition. Flexion (Flx) and Extension (Ext).

**Figure 2:**
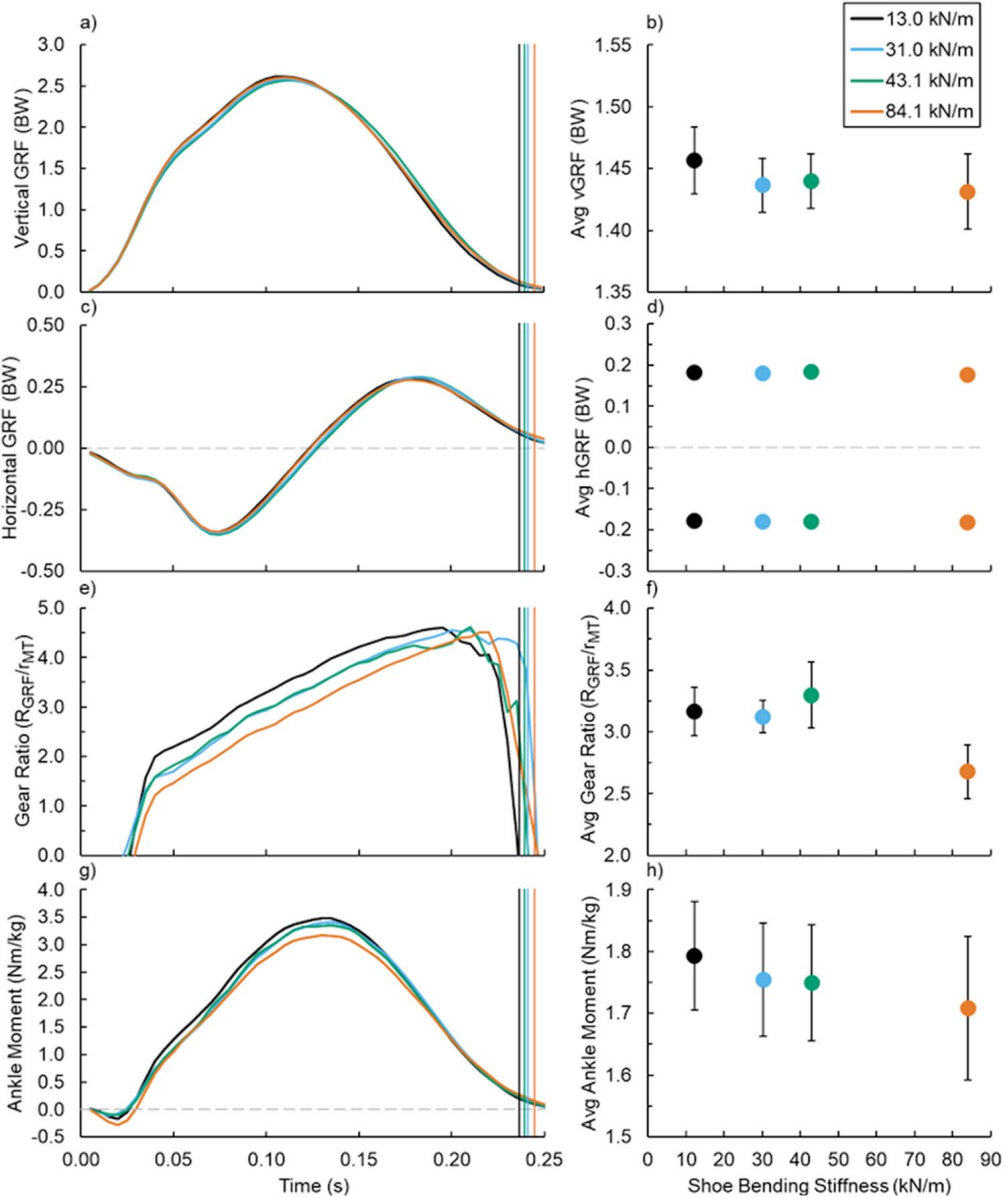
(Left) Average (**a**,**b**) vertical and (**c**,**d**) horizontal ground reaction force (GRF), (**e**,**f**) soleus muscle tendon (MT) gear ratio, and (**g**,**h**) net ankle moment versus time and (right) footwear 3-point bending stiffness (right): 13.0 (black), 31.0 (blue), 43.1 (green), and 84.1 kN/m (orange). Vertical lines indicate the average end of ground contact for the respective condition and error bars indicate SE when visible.

Footwear bending stiffness did not affect hip, knee, or ankle angles or moments (Fig. 1). Specifically, footwear bending stiffness was not associated with average, minimum, or maximum ankle (all p ≥ 0.121) (Fig. 1e and Fig. 2g,h), knee (all p ≥ 0.128) (Fig. 1c), or hip (all p ≥ 0.076) angle (Fig. 1a). Similarly, footwear bending stiffness did not affect average or maximum ankle (both p ≥ 0.060) (Fig. 1f), knee (both p ≥ 0.239) (Fig. 1d), or hip (both p ≥ 0.112) (Fig. 1b) moment.

### Stride kinematics and ground reaction forces

Increased footwear bending stiffness was associated with longer ground contact time (p = 0.048), but not step time (p = 0.956). Regarding GRFs, neither stance average vertical (p = 0.209) (Fig. 2a,b), braking (p = 0.441) (Fig. 2c,d), nor propulsive (p = 0.133) (Fig. 2c,d) GRF differed across footwear bending stiffness conditions. Additionally, footwear bending stiffness did not affect the fraction of vertical (p = 0.881) or horizontal (p = 0.816) GRF exhibited during the first half of ground contact.

### Muscle–tendon dynamics

**Figure 3:**
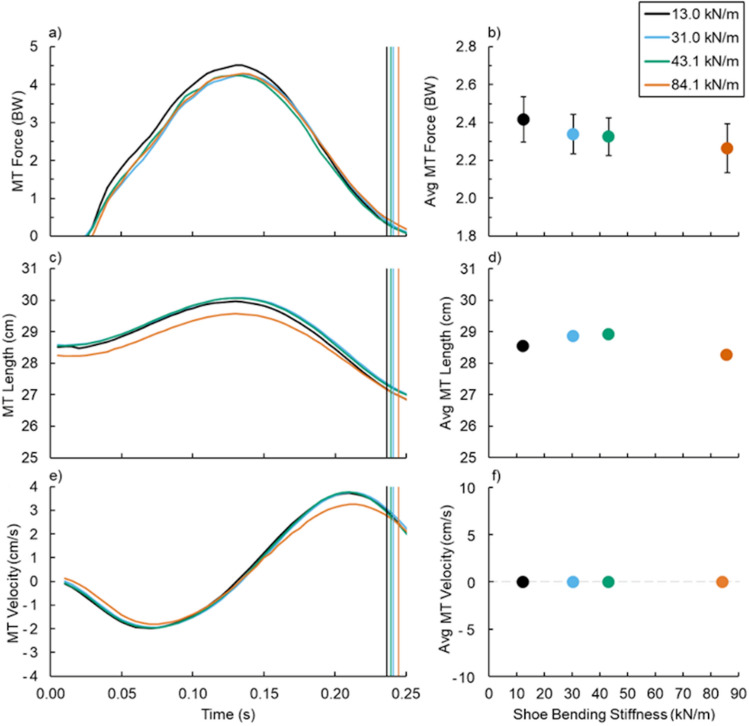
(Left) Average (**a**,**b**) soleus muscle–tendon (MT) force, (**c**,**d**) length, and (**e**,**f**) velocity versus time and (right) footwear 3-point bending stiffness (right): 13.0 (black), 31.0 (blue), 43.1 (green), and 84.1 kN/m (orange). Vertical lines indicate the average end of ground contact for the respective footwear condition and error bars indicate SE when visible.

Footwear bending stiffness did not affect soleus muscle–tendon (MT) dynamics (Fig. 3). Neither average soleus MT force (p = 0.080) (Fig. 3a,b), length (p = 0.150) (Fig. 3c,d), nor velocity (p = 0.719) (Fig. 3e,f) during ground contact changed with altered footwear bending stiffness. Additionally, the ratio of the GRF versus soleus MT moment arms to the ankle-joint center (gear ratio, also known as 1/effective mechanical advantage) was not affected by footwear bending stiffness (average and maximum gear ratio p = 0.371 and p = 0.752, respectively) (Fig. 2e,f).

### Soleus dynamics

**Figure 4:**
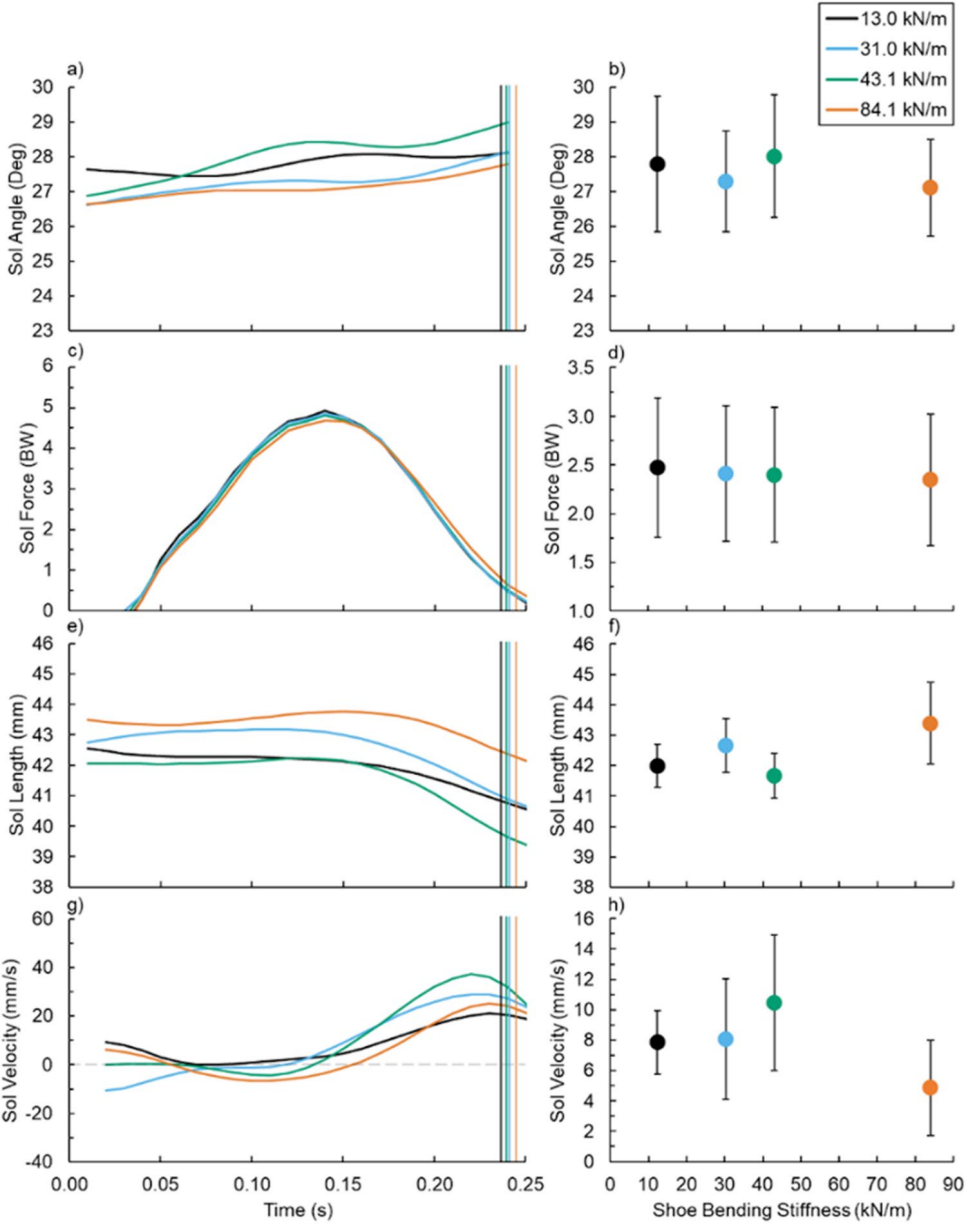
(Left) Average (**a**,**b**) soleus (Sol) fascicle angle, (**c**,**d**) force, (**e**,**f**) length, and (**g**,**h**) velocity versus time and (right) footwear 3-point bending stiffness (right): 13.0 (black), 31.0 (blue), 43.1 (green), and 84.1 kN/m (orange). Vertical lines indicate the average end of ground contact for the respective footwear condition and error bars indicate SE.

**Figure 5:**
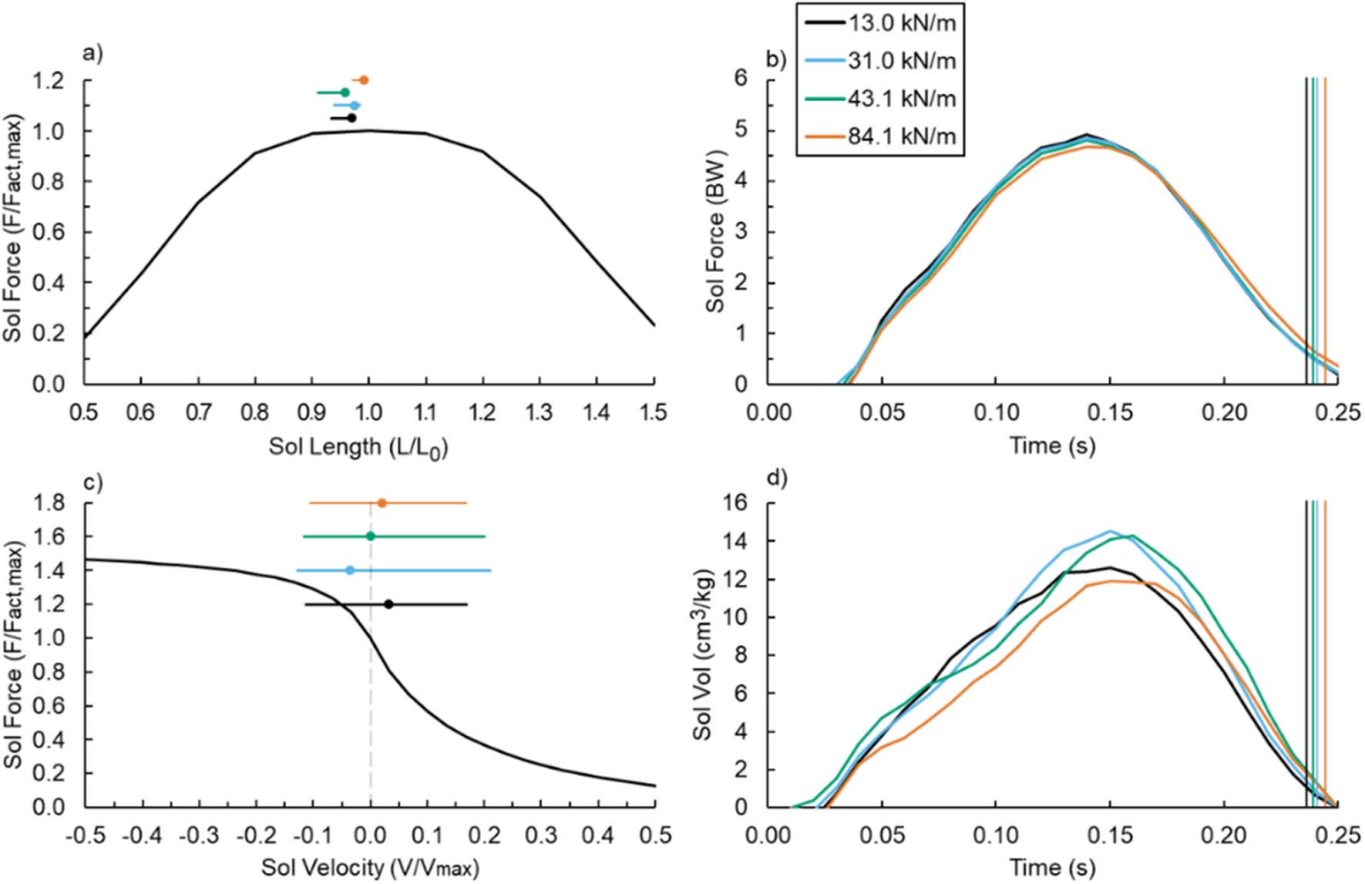
Estimated (**a**) Soleus (Sol) force–length and (**c**) force–velocity relationships during ground contact. The marker indicates soleus initial ground contact and the horizontal line indicates soleus operating range during ground contact. (**b**) Sol force and (**d**) volume (Vol) throughout ground contact and vertical lines indicate the average end of ground contact.

**Figure 6:**
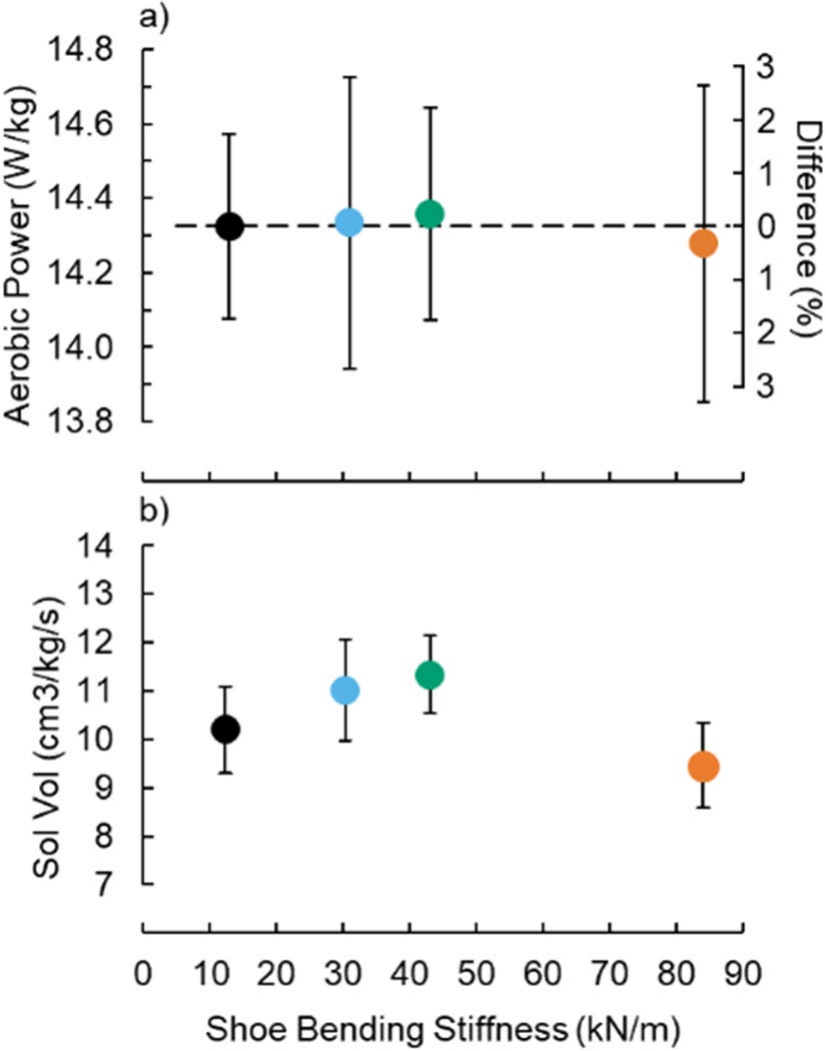
Average (± SE) (**a**) gross aerobic power and (**b**) activated soleus (Sol) volume (Vol) per stride. Right axis: Percent difference in the respective variables from the Adidas condition without a carbon fiber plate versus shoe bending stiffness.

Footwear bending stiffness did not influence average or maximum soleus fascicle pennation angle (both p ≥ 0.476) (Fig. 4a,b), force (both p ≥ 0.115) (Figs. 4c,d, 5b), length (p ≥ 0.286) (Fig. 4e,f and Fig. 5a), or velocity (both p ≥ 0.224) (Fig. 4g,h and Fig. 5c). As such, footwear bending stiffness did not affect stride-average soleus active muscle volume (p = 0.538; d = 0.241) (Figs. 5d, 6b).

### Muscle activation

**Figure 7:**
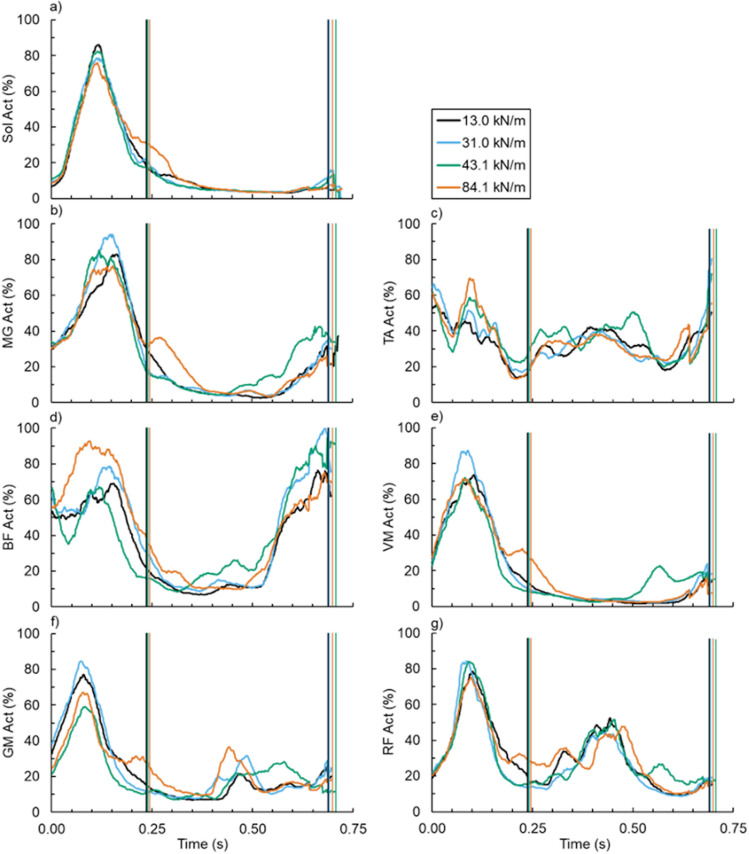
Average (**a**) soleus (Sol), (**b**) medial gastrocnemius (MG), (**c**) tibialis anterior (TA), (**d**) biceps femoris (BF), (**e**) vastus medialis (VM), (f) gluteus maximus (GM), and (**g**) rectus femoris (RF) versus time (left) across footwear 3-point bending stiffness: 13.0 (black), 31.0 (blue), 43.1 (green), and 84.1 kN/m (orange). Vertical lines indicate the average end of ground contact and stride for the respective footwear condition.

Footwear bending stiffness did not affect stance- or stride-averaged activation of any measured muscle: soleus (both p ≥ 0.315) (Fig. 7a), medial gastrocnemius (both p ≥ 0.538) (Fig. 7b), tibialis anterior (both p ≥ 0.445) (Fig. 7c), biceps femoris (both p ≥ 0.190) (Fig. 7d), vastus medialis (both p ≥ 0.146) (Fig. 7e), gluteus maximus (both p ≥ 0.603) (Fig. 7f), or rectus femoris (both p ≥ 0.406) (Fig. 7g) (Table 2).

**Table 2:** Stride averaged normalized muscle activation ± SD normalized to the respective muscle’s average maximum value during running with the Adidas (13.0 kN/m) footwear condition.

Footwear bending stiffness (kN/m)	Tibialis anterior (%)	Soleus (%)	Medial gastroc-nemius (%)	Vastus medialis (%)	Rectus femoris (%)	Biceps femoris (%)	Gluteus maximus (%)
13.0 ± 1.0	37 ± 7	24 ± 16	24 ± 5	19 ± 2	27 ± 10	40 ± 14	31 ± 15
31.0 ± 1.5	37 ± 9	21 ± 5	26 ± 3	20 ± 3	22 ± 12	39 ± 14	28 ± 9
43.1 ± 1.6	39 ± 9	21 ± 3	28 ± 4	19 ± 4	26 ± 12	38 ± 10	23 ± 6
84.1 ± 1.1	38 ± 9	20 ± 3	25 ± 3	20 ± 3	27 ± 13	40 ± 10	26 ± 7

### Running economy

Footwear bending stiffness did not affect gross aerobic power (p = 0.458; d = 0.04) (Fig. 6a). Only the 84.1 ± 1.1 kN/m footwear bending stiffness condition elicited a mean gross aerobic power value that was numerically less (non-significantly) than the footwear condition without a carbon fiber plate (13.0 ± 1.0 kN/m). Compared to the 13.0 ± 1.0 kN/m footwear condition, the 84.1 ± 1.1 kN/m footwear bending stiffness condition yielded 0.3 ± 2.2% lower gross aerobic power (paired t-test p = 0.663). To achieve a strong statistical power regarding the gross aerobic power elicited from the 84.1 ± 1.1 kN/m versus 13.0 ± 1.0 kN/m footwear bending stiffness condition (statistical power = 0.8), post-hoc analyses suggest that we would need to test 9104 participants.

Individually, the footwear condition that minimized running economy was 13.0 ± 1.0 kN/m for 1 participant, 31.0 ± 1.5 kN/m for 4 participants, 43.1 ± 1.6 kN/m for 4 participants, and 84.1 ± 1.1 kN/m for 6 participants. Also, the footwear bending condition that elicited the worst running economy was 13.0 ± 1.0 kN/m for 5 participants, 31.0 ± 1.5 kN/m for 3 participants, 43.1 ± 1.6 kN/m for 3 participants, and 84.1 ± 1.1 kN/m for 4 participants (Supplementary Fig. S1a–o).

## Discussion

Across a 6.4-fold increase in footwear bending stiffness, our participants ran with nearly identical body, limb-joint, and calf muscle mechanics, as well as elicited non-different running economy values. Footwear bending stiffness did not affect participant GRFs, limb-joint kinematics, or kinetics. Similarly, soleus MT and fascicle dynamics were unaltered across conditions. Regarding our hypotheses, running in stiffer footwear did not affect soleus fascicle force, length, or velocity; leading us to reject our initial hypothesis. While no previous study has quantified muscle fascicle dynamics from athletes running in shoes that varied in bending stiffness, our participant’s unaltered ankle-joint dynamics contrasts some previous reports^12,13,24^. Yet, the only biomechanical difference between our study and the classic investigation that reported that adding carbon fiber plates to shoe soles improve running economy^12^ is that the classic investigation found an increased maximum ankle moment with the use of stiffer footwear, whereas we did not. Further, while there are likely covariates, one previous study reported that athletes running in commercial shoes with curved carbon fiber plates embedded in their soles exhibited shorter GRF-ankle joint moment arms during ground contact compared to without carbon fiber plates^38^. Therefore, footwear with increased bending stiffness may not universally increase ankle-joint gear ratio.

Despite controlling for shoe mass, adding carbon fiber plates to footwear did not affect running economy nor soleus active muscle volume. Thus, we rejected our second hypothesis. Because footwear bending stiffness did not affect the stride-average activation for any of the measured muscles (Table 2, Fig. 7), none of the respective active muscle volumes changed across footwear conditions (active muscle volume = total muscle volume × relative activation)^37^. This is now the fourth study that failed to replicate Roy and Stefanyshyn’s classic investigation^12^, which stated that adding carbon fiber plates to shoe soles improves running economy^14–17^. Since the classic investigation, only Oh and Park^13^ reported that adding carbon fiber plates to running shoes elicited a relative footwear stiffness that improves running economy at 2.4 m/s. Moreover, the classic investigation^12^ reported that participant body mass was inversely correlated with the change in oxygen uptake at their intermediate footwear stiffness condition (38 kN/m) relative to footwear condition that did not have a carbon fiber plate (18 kN/m). Hence, compared to their smaller participants, the running economy of their larger participants improved more by adding carbon fiber plates to their shoe soles. In the present study, post-hoc analyses revealed that participant body mass was independent to the change in aerobic power during the most compliant footwear condition versus any of the stiffer footwear conditions (all p ≥ 0.502). Moreover, due to the implications of muscle dynamics on aerobic power^37^, we performed post-hoc linear regressions which revealed that the change in aerobic power from the footwear condition that did not contain a carbon fiber insole (13.0 ± 1.0 kN/m) was not correlated to the corresponding change in contact time (p = 0.135), soleus force generation (p = 0.614), or soleus velocity (p = 0.324). Further, there were two a potentially spurious weak correlations: (1) soleus active muscle volume versus gross aerobic power (r = -0.329; p = 0.039) and (2) change in soleus length versus change in gross aerobic power (r = 0.311, p = 0.040). Thus, we did not uncover any reasonable muscle-level parameters that correlated with the aerobic power when athletes ran in footwear conditions using carbon fiber plates versus without carbon fiber plates.

If footwear bending stiffness does not affect running economy, why does wearing Nike prototype footwear with carbon fiber plates embedded in their midsole (Nike) improve running economy compared to wearing Adidas footwear?^39^ Perhaps Nike’s carbon fiber plate provide the structure necessary for the midsole foam to function. Despite a 264% increased bending stiffness, when athletes run in Nike they elicit slightly shorter GRF to ankle-joint moment arms compared to running in Adidas footwear^38^. This increased footwear bending stiffness and shortened ankle-joint moment arm may be related to Nike’s curved carbon-fiber midsole plates^39^. Additionally, compared to the Adidas footwear, the respective Nike soles are ~ 8 mm taller (35–62% taller depending on midsole location), the midsole foam is roughly half as stiff (in-series linear stiffness, not bending), and its hysteresis is 11.1% less during vertical loading and unloading^39^. Altogether, because both decreased linear stiffness^40–42^ and relative mechanical energy dissipation^43^ in-series to the stance-limb are associated with more economical running, Nike footwear may elicit superior running economy values than Adidas footwear due to their relatively compliant and resilient midsole foam—not increased bending stiffness.

This study has potential limitations. First, our carbon fiber plates were located between the athlete’s sock and the Adidas midsole foam. The lack of cushioning on top of the stiffer carbon fiber plates may have elicited less comfortable footwear compared to the more compliant footwear conditions. Second, prior to the experimental trials, each participant performed a five-minute treadmill running habituation trial in the Adidas footwear without a carbon-fiber in-sole. Thus, differences in the habituation time between the footwear bending stiffness conditions may have affected our results. Even though humans adapt their biomechanics in just one step when landing onto terrain with different compliance^44–46^, running with carbon fiber insoles may require a more extensive habituation period, like that of more complicated lower-limb devices (e.g. exoskeletons)^47–49^. Additionally, we quantified soleus dynamics and not gastrocnemius dynamics because the soleus is the largest ankle plantar flexor^50^, it is the primary muscle that lifts and accelerates the participant’s center of mass during locomotion^51,52^, it likely generates the greatest muscle force of any plantar flexor^30^, and it is often estimated to consume the most metabolic energy of any plantar flexor during running^30,53,54^. Consistent with previous running studies that related longitudinal bending stiffness to metabolic energy expenditure^12,13^, we used a controlled laboratory environment and adequate sample size to relate metabolic energy expenditure collected in one session to biomechanical data collected from a separate session^55^. Moreover, regardless of how little footwear technology improves metabolic energy expenditure, even small improvements help separate champions from their peers in competitive athletics.

## Conclusion

Changing footwear bending stiffness hardly changes athlete biomechanics and may not improve running economy. Therefore, if competitive distance runners went back in time, added carbon fiber plates to their footwear, and re-raced, their performance would likely not change.

## Methods

### Participants

Fifteen males participated (Table 1). All participants were apparently free of cardiovascular, orthopedic, and metabolic disorders, and could run 5 km in < 25 min. Prior to the study, each participant gave informed written consent in accordance with the Georgia Institute of Technology Central Institutional Review Board. During the study. We followed the Georgia Institute of Technology Central Institutional Review Board’s approved protocol and carried out the study in accordance with these approved guidelines and regulations.

### Footwear

We acquired the Adidas Adizero Adios BOOST 2 (Adidas) running shoes in US men’s size 9, 10, 11, and 12. The Adidas are the same shoe model that Dennis Kimetto wore to set a previous marathon (42.2 km) world record (2:02:57 h:min:s). Next, we fabricated sets of custom carbon fiber in-soles that were 0.8, 1.6, and 3.2 mm thick to fit the Adidas shoes.

We characterized the 3-point bending stiffness of each shoe and in-sole condition following previously described methods^12,25,29^. Briefly, we performed 3-point bending tests by placing each footwear condition in a frame with two supporting bars 80 mm apart. We applied a vertical force to the top of each footwear condition midway between the two supporting bars, approximately where the foot’s metatarsophalangeal joint would be located using a materials testing machine (Instron, Norwood, MA, USA). We applied force three consecutive times to displace each shoe 10 mm following a 2 N preload (loading rate: 8 mm/s). We calculated footwear 3-point bending stiffness during loading using the average linear slope of the force–displacement data (100 Hz) from the following displacement range: 5 to 9 mm. We also set each athlete’s footwear mass equal to their largest footwear condition, which was the Adidas plus thickest carbon fiber in-sole. For example, the size 9 Adidas shoe is 199 g and its stiffest in-sole was 60 g. Accordingly, we set all size 9 footwear conditions to 259 g by securing mass to the tongue of each shoe.

### Protocol

Each participant completed two experimental sessions. During the first session (aerobic session), participants performed a 5-min standing trial followed by five 5-min treadmill (Bertec Corporation, Columbus, OH, USA) running trials at 3.5 m/s. Prior to each trial, participants rested for at least 5 min. The first running trial served as habituation to treadmill running in the Adidas footwear (no carbon fiber in-sole). During each subsequent trial, participants ran using a different footwear condition: Adidas as well as Adidas with 0.8, 1.6, and 3.2 mm thick carbon fiber in-soles. We randomized footwear trial order. Each participant’s second session (biomechanics session) occurred at the same time of day and < 10 days following their first session. During the second session, participants performed four 2-min treadmill running trials at 3.5 m/s using the same footwear conditions as the first session in a re-randomized order. We performed separate aerobic and biomechanics sessions to mitigate the potential for technical difficulties to arise by measuring biomechanics over a briefer session than needed for accurate metabolic measurements.

### Aerobic energy expenditure

We asked participants to arrive to their aerobic session 3-h post-prandial. Throughout each of the aerobic session’s trials, we used open-circuit expired gas analysis (TrueOne 2400, ParvoMedic, Sandy, UT, USA) to record the participant’s rates of oxygen uptake (V̇o_2_) and carbon dioxide production (V̇co_2_). We monitored each participant’s respiratory exchange ratio (RER) throughout each trial to ensure that everyone primarily relied on aerobic metabolism during running; indicated by an RER ≤ 1.0^31^. Next, we averaged V̇o_2_ and V̇co_2_ over the last 2-min of each trial and used a standard equation^56^ to calculate aerobic power (W). Subsequently, we subtracted the corresponding session’s standing aerobic power (Table 1) from each running trial and divided by participant mass to yield mass-normalized aerobic power (W/kg).

### Biomechanics

Prior to the biomechanics session’s running trials, we placed reflective markers on the left and right side of each athlete’s lower body following a modified Helen Hayes marker set: superficial to the head of the 1st and 5th metatarsal, posterior calcaneus, medial and lateral malleoli, lateral mid-shank, medial and lateral knee-joint center, lateral mid-thigh, greater trochanter, anterior superior iliac crest, posterior superior iliac crest, and superior iliac crest. During the ensuing trials, we recorded vertical and anterior–posterior GRFs (1000 Hz) as well as motion capture (200 Hz) data during the last 30 s of each trial. We performed a fast fourier transform on the raw GRF data from six random participants and then filtered the raw GRFs and center-of-pressure data appropriately: using a fourth-order low-pass critically damped filter (14 Hz)^54,57,58^. We filtered motion capture using a fourth-order low-pass Butterworth filter (7 Hz)^57,59–62^. Using the filtered GRFs, we calculated whole-body stride kinematics (stance and stride time) and GRF parameters (stance average vertical and resultant GRF, as well as mean braking and propulsive horizontal GRFs^63^) with a custom MATLAB script (Mathworks, Natick, MA) that detected periods of ground contact using a 30 N vertical GRF threshold. We categorized each participant as a heel striker or mid/forefoot striker based on visual inspection and whether their vertical GRF trace had an impact peak or not (Table 1). If the participant visually appeared to contact the ground with their heel and displayed a vertical GRF impact peak they were deemed a heel striker^64^. Participants that did not satisfy these criteria were deemed a mid/forefoot strikers.

We performed inverse dynamics and determined limb joint kinematics (limb joint angles and GRF-to-joint-center moment arms) and kinetics (limb joint moments) (C-motion Inc., Germantown, MD; Mathworks Inc., Natick, MA, USA). Subsequently, we computed each participant’s instantaneous soleus muscle–tendon (MT) moment arm, length, velocity, and force. We used participant anthropometric data and limb-joint angles to calculate the respective soleus MT length^36^, velocity, and moment arm^36,65^. Next, we used each soleus MT moment arm (r) and net ankle-joint moment (M) to calculated soleus MT force (F) by deeming that the soleus generates 54% of total plantar flexor force based on its relative physiological cross sectional area^66^.

Prior to the biomechanics session’s trials, we secured a linear-array B-mode ultrasound probe (Telemed, Vilnius, Lituania) to the skin superficial of each athlete’s right soleus. Using ultrasonography, we recorded mid-soleus fascicle images (100 Hz) during at least five consecutive strides per trial. We processed the images using a semi-automated tracking software^67^ to determine instantaneous soleus pennation angle and fascicle length. For semi-automated images that did not accurately track the respective soleus fascicle angle and/or length, we manually redefined the respective fascicle’s parameters. We used soleus MT force and fascicle angle to calculate soleus fascicle force, length, and velocity in congruence with previous studies^37,57^. We filtered soleus fascicle angle and length using a fourth-order low-pass Butterworth filter (10 Hz) and took the derivative of fascicle length with respect to time to determine fascicle velocity. Subsequently, we determined relative soleus fascicle length and velocity by deeming that soleus fascicles are at 97% of their optimal length at initial ground contact in the Adidas condition^32^ and that their maximum velocity is 6.77 L_0_/s^53^, respectively. We deemed average ± SD maximum soleus velocity to equal 297.1 ± 16.5 mm/s. Due to technical difficulties, we were unable to compute accurate active soleus volume during 18 of 60 trials; spanning 5 participants.

We recorded surface EMG signals from the biomechanics session’s running trials using the standard procedures of the International Society for Electrophysiology and Kinesiology^68^. Prior to the first trial, we shaved and lightly abraded the skin superficial to the medial gastrocnemius, soleus, tibialis anterior, vastus medialis, rectus femoris, biceps femoris, and gluteus maximus of each participant’s left leg with electrode preparation gel (NuPrep, Weaver and Co., Aurora, CO). Next, we placed a bipolar surface electrode (Delsys Inc., Natick, MA) over the skin superficial to each respective muscle belly and in the same orientation as the respective muscle fascicle. We recorded EMG signals at 1000 Hz and verified electrode positions and signal quality by visually inspecting the EMG signals while participants contracted the respective muscle. Based on visual inspection and technical difficulties, we removed 97 of 420 potential muscle activation signals due to their poor signal quality; spanning 4 participants. To analyze EMG signals from the running trials, we band-pass filtered the raw EMG signals to retain frequencies between 20 and 450 Hz, full-wave rectified the filtered EMG signals, and then calculated the root mean square of the rectified EMG signals with a 40 ms moving window^69,70^. Lastly, we normalized each muscle activation to the average maximum activation of the respective muscle during running in the Adidas condition sans carbon fiber plates^70^.

### Statistics

An a priori analysis on Roy and Stefanyshyn’s data^12^, suggested that fifteen participants would achieve a strong statistical power (0.895) between footwear bending stiffness and metabolic power. We performed a linear regression on the footwear’s force–displacement profile, which was measured from a materials testing device. We performed independent repeated measures ANOVAs to determine whether footwear bending stiffness (independent variable) affected athlete running biomechanics (hip, knee, and ankle stance average, minimum, and maximum angle; hip, knee, and ankle stance average and maximum moment; ground contact time; step time; stance average vertical, braking, and propulsive GRF; fraction of vertical and horizontal GRF during the first half of stance; stance average muscle–tendon force, length, velocity, and gear ratio; stance average and maximum soleus fascicle pennation angle, force, length, velocity; stance average, stride average soleus active muscle volume; stance average and stride average soleus, medial gastrocgnemius, tibialis anterior, biceps femoris, vastus medialis, gluteus maximus, and rectus femoris; and gross aerobic power (dependent variables). We presented cohen’s d effect size for gross metabolic power and stride average soleus active muscle volume. We performed all statistical tests using R-studio (R-Studio Inc., Boston, USA) and G*Power software.

## Supplementary information

Supplementary Information.
