# Supplementary material for: Adding carbon fiber to shoe soles may not improve running economy: a muscle-level explanation
Source: Sci Rep. 2020 Oct 13;10:17154. doi: 10.1038/s41598-020-74097-7 (PMC7555508; doi:10.1038/s41598-020-74097-7)
Supplement: Supplementary file 1 — Supplementary Information. [file 41598_2020_74097_MOESM1_ESM.docx]

**Title:** Adding carbon fiber to shoe soles may not improve running economy:

a muscle-level explanation

**Authors:** Owen N. Beck,^1,2*^ Pawel R. Golyski,^1,3^ & Gregory S. Sawicki^1,2,3^

^1^George W. Woodruff School of Mechanical Engineering, ^2^School of Biological Sciences,

and ^3^Parker H. Petit Institute for Bioengineering and Biosciences,

Georgia Institute of Technology, Atlanta, GA

***Corresponding Author**

Name: Owen N. Beck

Address: Georgia Institute of Technology

School of Mechanical Engineering

455 Callaway Manufacturing Research Center Building

813 Ferst Dr NW

Atlanta, GA 30332

E-mail: [obeck3@gatech.edu](mailto:obeck3@gatech.edu)

**Supplementary Table 1.** Number of participants that yielded the lowest and highest value for select biomechanical variables.

| Footwear Bending Stiffness (kN/m) | Ground Contact  Time | | Hip Moment | | Knee Moment | | Ankle Moment | | Soleus Force | | Soleus Fascicle Length* | | Soleus Fascicle Velocity* | |
| --- | --- | --- | --- | --- | --- | --- | --- | --- | --- | --- | --- | --- | --- | --- |
|  | Low | High | Low | High | Low | High | Low | High | Low | High | Low | High | Low | High |
| 13.0 ± 1.0 | 6 | 1 | 7 | 1 | 6 | 3 | 1 | 8 | 4 | 7 | 1 | 3 | 4 | 2 |
| 31.0 ± 1.5 | 3 | 4 | 1 | 4 | 3 | 4 | 6 | 2 | 4 | 2 | 2 | 3 | 1 | 4 |
| 43.1 ± 1.6 | 3 | 3 | 4 | 4 | 1 | 3 | 4 | 1 | 4 | 3 | 5 | 1 | 2 | 4 |
| 84.1 ± 1.1 | 3 | 7 | 3 | 6 | 5 | 5 | 4 | 4 | 3 | 3 | 3 | 4 | 4 | 1 |

*n=11 participants due to technical difficulties.


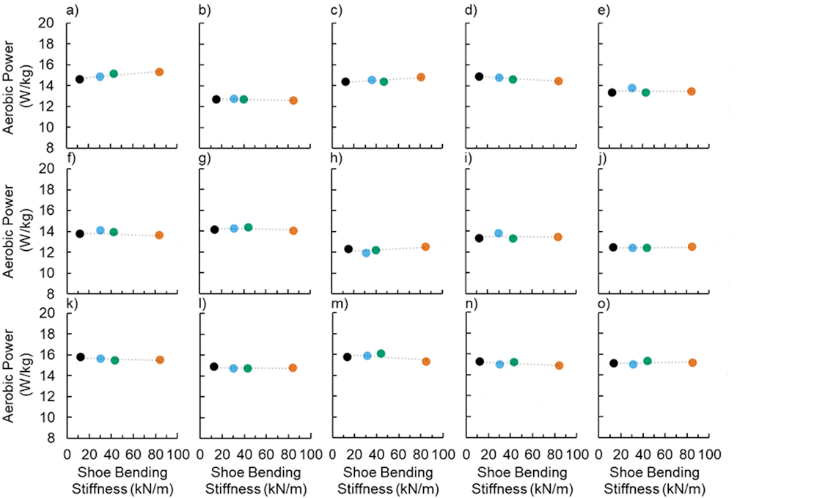


**Supplementary Figure 1.** Gross aerobic power versus footwear bending stiffness for each participant.
